# Ascomylactam C Induces an Immunogenic Cell Death Signature via Mitochondria-Associated ER Stress in Lung Cancer and Melanoma

**DOI:** 10.3390/md21120600

**Published:** 2023-11-21

**Authors:** Yun Huang, Hongmei Yan, Bingzhi Zhang, Ge Zhu, Jianchen Yu, Xuhan Xiao, Wenxuan He, Yan Chen, Xiaoxia Gao, Zhigang She, Mengfeng Li, Jie Yuan

**Affiliations:** 1 School of Basic Medical Sciences, Southern Medical University, Guangzhou 510515, China; wyhl2014@163.com (Y.H.); yanhongmei126@i.smu.edu.cn (H.Y.); 2 Key Laboratory of Tropical Disease Control (Sun Yat-sen University), Ministry of Education, Guangzhou 510080, China; zhug6@mail2.sysu.edu.cn (G.Z.); yujchen@mail2.sysu.edu.cn (J.Y.); xiaoxh26@mail2.sysu.edu.cn (X.X.); 3 School of Pharmacy, Guangdong Pharmaceutical University, Guangzhou 510006, China; bingzhi0908@163.com (B.Z.); gaoxxia91@163.com (X.G.); 4 Zhongshan School of Medicine, Sun Yat-sen University, Guangzhou 510080, China; 5 School of Life Sciences and Biopharmaceutics, Guangdong Pharmaceutical University, Guangzhou 510006, China; 13078435107@139.com; 6 Department of Traditional Chinese Medicine, School of Pharmacy, Anhui Medical University, Hefei 230032, China; cychemistry@163.com; 7 School of Chemistry, Sun Yat-sen University, Guangzhou 510275, China; cesshzhg@mail.sysu.edu.cn

**Keywords:** ascomylactam C, lung cancer, melanoma, endoplasmic reticulum stress, oxidative stress, immunogenic cell death

## Abstract

Ascomylactam C (AsC) is a new 13-membered-ring macrocyclic alkaloid, which was first isolated and identified in 2019 from the secondary metabolites of the mangrove endophytic fungus *Didymella* sp. CYSK-4 in the South China Sea. AsC has been found to have a broad-spectrum cytotoxic activity. However, the antitumor effects in vivo and mechanisms of AsC remain unclear. The aim of this study was to describe the effects of AsC on lung cancer and melanoma cells and to explore the antitumor molecular mechanism of AsC. In vitro, we used plate colony formation experiments and demonstrated the ability of AsC to inhibit low-density tumor growth. An Annexin V/PI cell apoptosis detection experiment revealed that AsC induced tumor cell apoptosis. In vivo, AsC suppressed the tumor growth of LLC and B16F10 allograft significantly in mice, and promoted the infiltration of CD4^+^ T and CD8^+^ T cells in tumor tissues. Mechanistically, by analyses of Western blotting, immunofluorescence and ELISA analysis, we found that AsC increased ROS formation, induced endoplasmic reticulum (ER) stress, activated the protein kinase RNA-like ER kinase (PERK)/eukaryotic translation initiation factor (eIF2α)/activating transcription factor 4 (ATF4)/C/EBP homologous protein (CHOP) signaling pathway, and induced immunogenic cell death (ICD) of tumor cells. Our results suggest that AsC may be a potentially promising antitumor drug candidate.

## 1. Introduction

Immunogenic cell death (ICD) is a specific type of cell death that can be induced by certain anticancer chemotherapeutics. Tumor cells undergoing ICD can induce an adaptive anticancer immune response [1]. Apoptotic cells display calreticulin protein (CRT) on their surfaces and secrete adenosine triphosphate (ATP) after undergoing ICD. Tumor cell release or exposure to danger-associated molecular patterns (DAMPs), such as CRT and ATP, can stimulate the attraction, activation and maturation of dendritic cells and eventually the antigen-specific priming of cytotoxic T lymphocytes (CTLs) [2]. The phosphorylation of the eukaryotic translation initiation factor (eIF2α) and the transcriptional activation of activating transcription factor 4 (ATF4) are the pathognomonic characteristics of ICD [3]. Recent studies have revealed that certain anticancer drugs, including oxaliplatin [4] and crizotinib [5], foster ICD and induce immune response against tumor growth. Interventions targeting ICD not only directly induce cancer cell death but also trigger antitumor immune responses, and these are promising for the formulation of anticancer strategies.

Reactive oxygen species (ROS) have been demonstrated to play an essential role in the ICD induced by anticancer drugs [6]. ROS are highly associated with damage to both the mitochondria and endoplasmic reticulum (ER). Furthermore, this leads to the upregulation of phosphorylated protein kinase RNA-like ER kinase (p-PERK), and phosphorylated eIF2α, thereby stopping CAP-dependent mRNA translation and favoring the translation of the mRNA coding for activating transcription factor 4 (ATF4). And then ATF4 induce C/EBP homologous protein (CHOP) transcription [3,7,8,9]. CHOP plays an important role in ER stress-induced apoptosis by regulating the expression of many antiapoptotic and pro-apoptotic genes. CHOP inhibited antiapoptotic genes include Bcl-2, Bcl-XL and Mcl-1, pro-apoptotic genes include Bim and Bax [10]. Moreover, ER stress can release numerous DAMPs, such as CRT and ATP into the cytoplasm, eventually leading to ICD [11,12].

Natural products and their structural analogues have become the main source of antitumor drugs. According to statistics, 206 of the 321 anticancer drugs approved from 1946 to 2019 are related to natural products [13]. Ascomylactams C (AsC) was isolated from the mangrove endophytic fungus *Didymella* sp. CYSK-4, which is a new alkaloid constructed of a 6/5/6/5 tetracyclic core modified with an additional 13-membered-ring macrocyclic moiety. Our previous studies found that AsC has cytotoxic effects in a variety of cancer cell models [14]. However, the anticancer mechanism of AsC remains unclear. In the present study, we found that AsC could inhibit the proliferation of lung cancer and melanoma cells in vitro and in vivo. Further studies showed that AsC increased the accumulation of reactive oxygen species (ROS) and caused the damage of mitochondria-associated endoplasmic reticulum (ER) membrane (MAM) structure. Furthermore, we detected the upregulation of the phosphorylation of PERK and eukaryotic initiation factor 2α (eIF2α) and the expression of the transcription factor CHOP, thereby initiating the unfolded protein response (UPR) in the cancer cells under the treatment of AsC. These results indicated that AsC promoted dendritic cell (DC) maturation in a ROS-dependent manner.

## 2. Results

### 2.1. AsC Reduces Lung Cancer and Melanoma Cell Viability In Vitro

**Figure 1:**
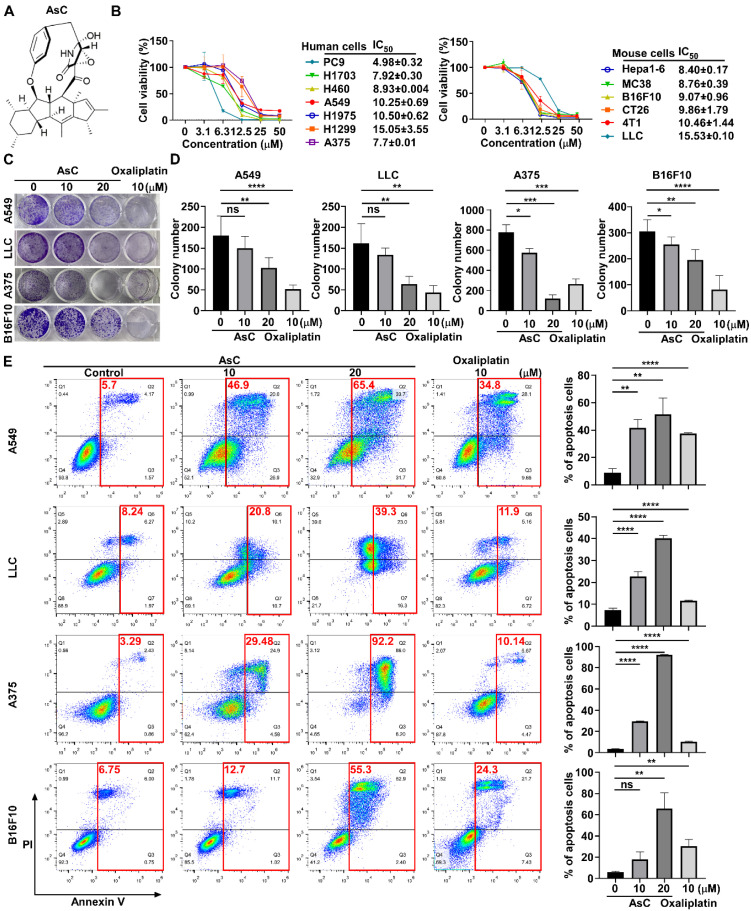
AsC inhibits the growth of cancer cells and induces apoptosis in vitro. (**A**) Chemical structure of AsC. (**B**) Cell viability of a variety of human lung cancer cells and mouse cancer cells shown in the figure treated by AsC for 48 h detected by 3-(4,5-dimethylthiazol-2-yl)-2,5-diphenyl tetrazolium bromide (MTT) assays. The bar shown represents the mean ± SD of samples from two independent experiments. (**C**) Clone formation efficiency of the cancer cells treated by AsC. A549, LLC, A375 and B16F10 cells were incubated with AsC or oxaliplatin at indicated concentrations in plates for 10 days. (**D**) The number of colonies was counted. Data are presented as mean ± SD. * *p* < 0.05, ** *p* < 0.01, *** *p* < 0.001, **** *p* < 0.0001, “ns” indicates not significant. (**E**) A549, LLC, A375 and B16F10 cells were treated with AsC or oxaliplatin at indicated concentrations for 24 h and Annexin V and PI staining cells were analyzed by flow cytometry. ** *p* < 0.01, **** *p* < 0.0001, “ns” indicates not significant.

To determine the effect of AsC (Figure 1A) on different cancer types, we tested the anticancer activity of AsC in human lung cancer cells (PC9, H1703, H460, A549, H1975 and H1299), human melanoma cells (A375 cell) and mouse cancer cells (LLC, Hepa1-6, MC38, B16F10, CT26 and 4T1) by 3-(4,5-dimethylthiazol-2-yl)-2,5-diphenyl tetrazolium bromide (MTT) assay, and the IC_50_ values were from 4.98 to 15.05 μM (Figure 1B). And we selected four commonly used lung cancer (A549, LLC) and melanoma cell lines (A375 and B16F10) as the cell models for further research. In addition, we also tested the antitumor activity of the clinical antitumor drug, oxaliplatin, in A549, LLC, A375 and B16F10 cells as the control, and the IC_50_ values were from 13.0 to 59.6 μM (Supplementary Materials Figure S1). To further explore the effect of AsC on the growth of cancer cells, we performed a colony formation assay to detect the effect of AsC or oxaliplatin on low density growth under the same treatment conditions. Based on the results of the cell viability assay and the growth rate of cell colonies, we treated the cells with indicated concentrations of AsC or oxaliplatin for 10 days (Figure 1C,D). The results showed that AsC and oxaliplatin could significantly inhibit the cancer cell proliferation in vitro, and the inhibitory of AsC was in a dose-dependent manner.

In order to elucidate whether the reduced proliferation of cancer cells would be due to the cytostatic or cytotoxic properties of AsC treatment, we evaluated the apoptosis of cancer cells under the treatment of AsC or oxaliplatin under equivalent treatment conditions by flow cytometry. According to the cell viability assay results and the morphological changes of cells after treatment with drugs or compounds, we treated cancer cells with indicated concentrations of AsC or oxaliplatin for 24 h and incubated them with Annexin V-APC and propidium iodide (PI). The results showed that AsC treatment strongly induced apoptotic response compared to the untreated control, resulting in an increased percentage of apoptotic cells (from 5.7% in untreated cells to 46.9%, 65.4% in treatment A549 cells with AsC 10, 20 μM). Similar experiments were performed in LLC, A375 and B16F10 cells and revealed similar results. In addition, the ability of AsC to induce apoptosis under the same treatment conditions was slightly stronger than that of oxaliplatin (Figure 1E). The results suggested that AsC inhibited lung cancer and melanoma cell proliferation and induced apoptosis.

### 2.2. AsC Suppresses Lung Cancer and Melanoma Cell Viability In Vivo

**Figure 2:**
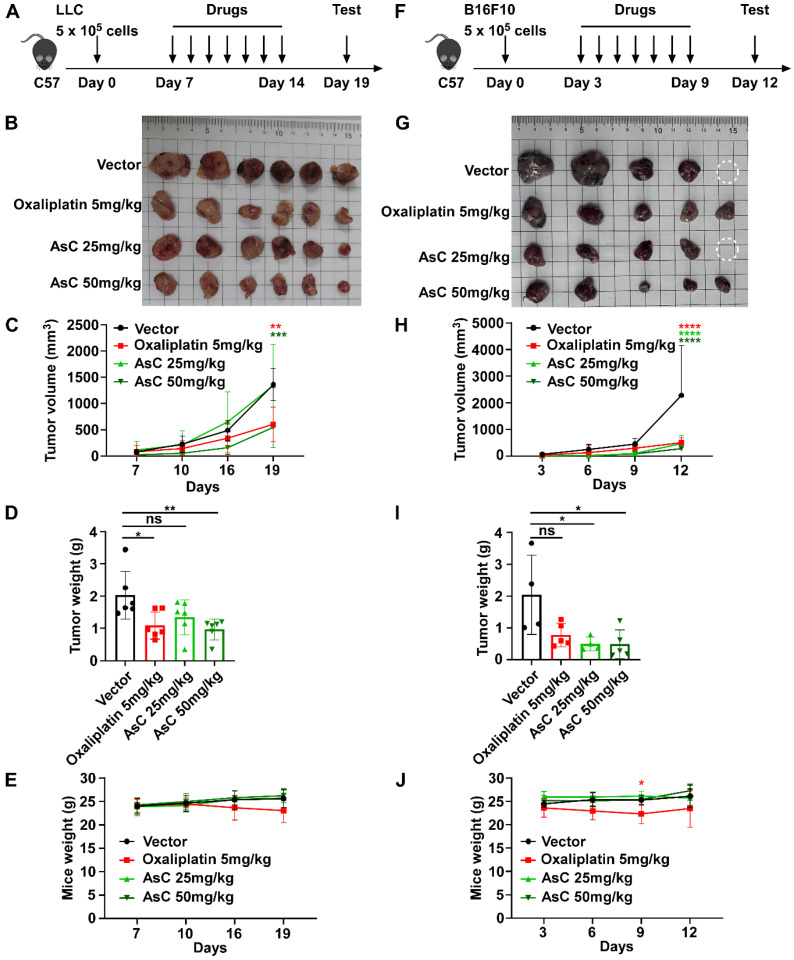
AsC inhibits the growth of melanoma and lung cancer cells in vivo. (**A**,**F**) Flowchart of the experimental design for the antitumor activity of AsC. (**B**,**G**) Subcutaneous tumors formed by the indicated cells were dissected and imaged. The circles represent tumors that were not obtained because the mice died before the end of the experiment. (**C**,**H**) Tumor volumes of LLC and B16F10 xenografts following treatment with AsC (25 mg/kg, 50 mg/kg), oxaliplatin (5 mg/kg) once every three days. The average volume and standard deviation are plotted, the statistical comparison vs. vehicle-treated control is shown by *t*-test. ** *p* < 0.01, *** *p* < 0.001, **** *p* < 0.0001. (**D**,**I**) The tumor weight of each group of mice. * *p* < 0.05, ** *p* < 0.01, “ns” indicates not significant. (**E**,**J**) The body weight change curves of each group of mice. * *p* < 0.05.

To investigate the anticancer activity of AsC in vivo, we used C57 mice to construct a mouse lung cancer and melanoma (LLC and B16F10) subcutaneous homograft cancer model. In each tumor model, mice were randomly divided into four groups and treated with vehicle, oxaliplatin (5 mg/kg), and AsC (25 mg/kg or 50 mg/kg) once every 3 days (Figure 2A,F). The results showed that both the AsC low-dose (25 mg/kg) and high-dose groups (50 mg/kg) could significantly inhibit the increase in tumor volume and tumor weight (Figure 2B,D,G,I). AsC had no significant effect on the body weight of mice, while the body weight of mice in the oxaliplatin group was significantly reduced (Figure 2E,J). In addition, in order to further observe the impact of AsC on various organs and tissues of mice, we performed H&E staining on the small intestine, kidney, liver and other tissues of mice in each treatment group. The results showed that the villus tissue of the mouse small intestine was destroyed in the oxaliplatin group. Severe pathological changes such as damaged glomerular integrity and severe hepatocyte edema were observed. However, in the AsC-treated groups, no other visible tissue damage was observed except for edema in the liver tissue (Supplementary Materials Figure S2). Furthermore, one mouse died in the vehicle control group and AsC low-dose group. Our results showed that AsC could inhibit the growth of lung cancer and melanoma cells with fewer side effects in vivo.

### 2.3. AsC Increases Infiltration of CD8^+^ T Cells in Lung Cancer and Melanoma Tissues

**Figure 3:**
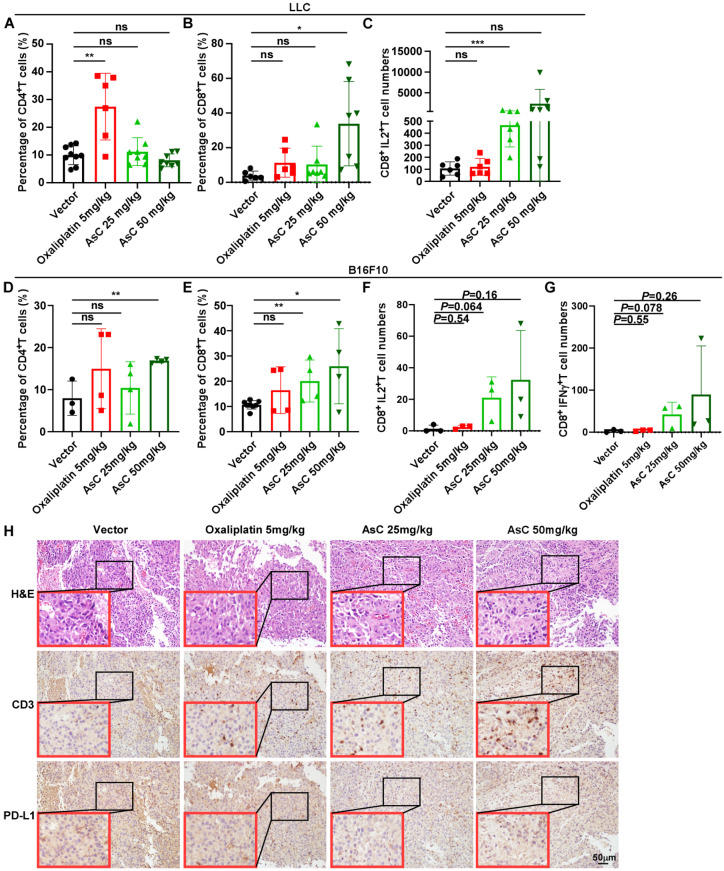
AsC promotes the infiltration of CD8^+^ T cells and reduces the expression of PD-L1 in vivo. (**A**–**F**) Isolation of LLC and B16F10 mouse tumor cells for flow cytometry staining. (**A**,**D**) Proportion of CD4^+^ T cells in LLC and B16F10 tumor tissues. (**B**,**E**) Proportion of CD8^+^ T cells in LLC and B16F10 tumor tissues. (**C**,**F**) The numbers of IL2^+^ T cells in CD8^+^ T cells in LLC and B16F10 tumor tissues. (**G**) The numbers of IFN-γ^+^ T cells in CD8^+^ T cells in B16F10 tumor tissues. The statistical comparison vs. vehicle-treated control is shown by *t*-test. * *p* < 0.05, ** *p* < 0.01, *** *p* < 0.001, “ns” indicates not significant. (**H**) Hematoxylin and Eosin (H&E) staining and immunohistochemistry (IHC) staining of CD3 and PD-L1 in B16F10 tumor tissues. The red boxes represent the images of the tumor tissue magnified 4 times by the black boxes (magnification, 200×).

Previous studies reported that interleukin 2 (IL2) could play multiple roles during the generation of effector and memory CD8^+^ T cell responses. CD8^+^ T cells intrinsically expressing IL2 possess stem cell properties and resist exhaustion and generate a memory phenotype [15]. Engineered IL2 partial agonists promote the expansion of CD8^+^ T cells and exhibits potent antitumor activity in vivo [16]. In addition, autocrine production of interferon-γ (IFN-γ) by CD8^+^ T cells can enhance its cytotoxic function, motility and cell survival, and promote the killing effect of CD8^+^ T cells [17]. In order to observe whether AsC can change the tumor immune microenvironment during the inhibition of lung cancer and melanoma, we examined the proportions of various immune cells in mouse tumor tissue cells, including CD4^+^ T cells, CD8^+^ T cells and Natural Killer (NK) cells. The results showed that the proportion of CD8^+^ T cells increased in both lung cancer and melanoma tumor tissues, while CD4^+^ T cells only increased in melanoma tissues, and the same phenomenon was not observed in lung cancer tissues. Meanwhile, more IL2 and IFN-γ were detected in CD8^+^ T cells in the AsC administration group (Figure 3A–G, Supplementary Materials Figures S3 and S4). In addition, no obvious changes were seen in NK cells in LLC (Supplementary Materials Figure S5).

In addition, to investigate the infiltration of lymphocytes in melanoma tumor tissue, we observed the expression and distribution of CD3^+^ T cells in melanoma tumor tissue through immunohistochemistry experiments. The results showed that the expression of CD3^+^ T in the AsC-treated group was significantly increased compared with the vehicle control group. Interestingly, the expression of Programmed death ligand 1 (PD-L1) was also reduced in the AsC-treated group. The above results show that AsC can promote the infiltration of CD8^+^ T cells in tumor tissues and reduce the expression of PD-L1 (Figure 3H).

### 2.4. AsC Induces ROS Accumulation and Mitochondrial Membrane Depolarization in Lung Cancer and Melanoma Cells

**Figure 4:**
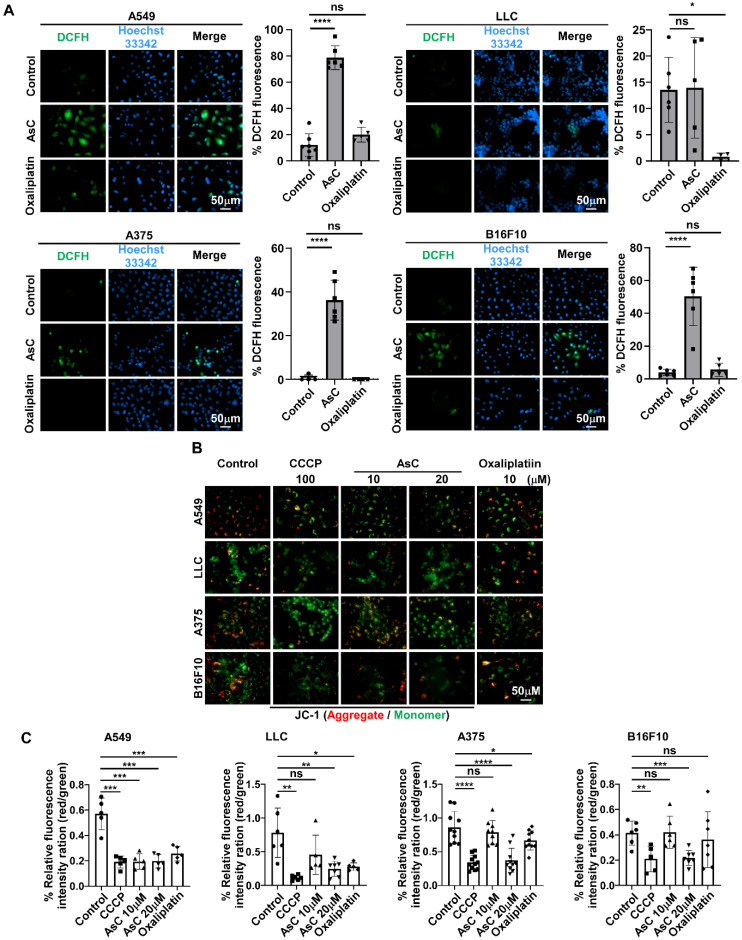
AsC promotes ROS accumulation and induces mitochondrial membrane depolarization in lung cancer and melanoma cells. (**A**) Fluorescence images of lung cancer (A549 and LLC) and melanoma (A375 and B16F10) cells stained with DCFH-DA and Hoechst33342 after treated with 10 μM AsC and oxaliplatin for 12 h. * *p* < 0.05, **** *p* < 0.0001, “ns” indicates not significant, (magnification, 400×). (**B**) Immunofluorescence images of ΔΨm alterations and (**C**) statistical analysis of fluorescence intensity in A549, LLC, A375 and B16F10 cells after different treatments for 6 h. * *p* < 0.05, ** *p* < 0.01, *** *p* < 0.001, **** *p* < 0.0001, “ns” indicates not significant.

In previous studies, we found that AsC has the effect of transforming “cold tumors” into “hot tumors”. Therefore, we speculated whether AsC could induce the occurrence of ICD. A large number of studies have shown that ROS and changes in mitochondrial function play a key role in the occurrence of ICD. ROS has been reported to trigger ICD and subsequently lead to anticancer immune responses [18,19,20]. Since ROS often serve as upstream signals for cell death [21,22], here we detected the ROS generation in lung cancer and melanoma cells by utilizing a DCFH-DA fluorescent dye. As shown in Figure 4A, after 12 h of AsC treatment, intracellular ROS production increased significantly in A549, A375 and B16F10 cells, but there was no significant change in LLC cells. Furthermore, just as previous reported, mitochondrial function was evaluated by assessing mitochondrial membrane potential (ΔΨm) using a JC-1 kit (Dojindo Molecular Technologies, Tokyo, Japan) [21]. The cancer cells were treated with indicated concentrations of CCCP, AsC and oxaliplatin for 6 h. And the results showed that with increasing treatment concentration of AsC, ΔΨm was significantly reduced in LLC, A375 and B16F10 cells, except A549 cells (Figure 4B,C). The above results indicate that AsC could cause the increase in intracellular ROS production and the damage to the mitochondrial function. However, the mechanism of this damage is complex because the phenomena are not entirely consistent in different cells.

### 2.5. AsC Induces DAMPs and ICD in Lung Cancer and Melanoma Cells

**Figure 5:**
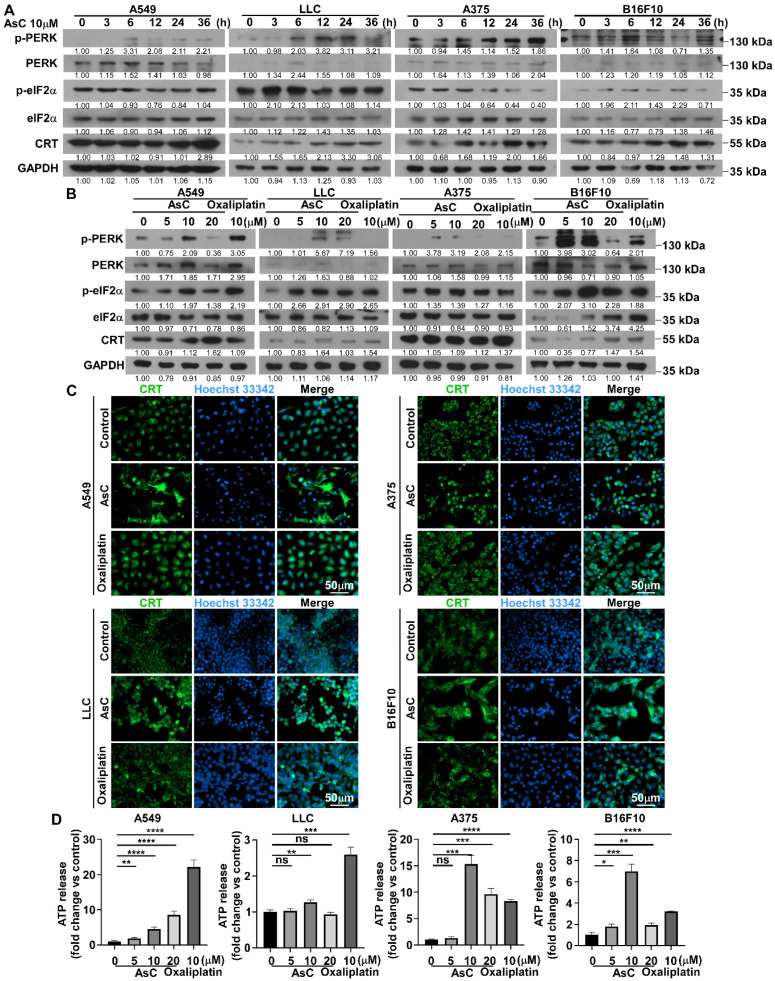
UPR and DAMPs released upon ER stress following AsC treatment. (**A**) A549, LLC, A375 and B16F10 cells were treated by AsC at indicated concentrations for different times (0, 3, 6, 12, 24, 36 h). Levels of p-PERK, PERK, p-eIF2α, eIF2α and CRT proteins were detected using Western blotting. (**B**) A549, LLC, A375 and B16F10 cells were treated with 10 μM AsC or oxaliplatin for 24 h. Levels of p-PERK, PERK, p-eIF2α, eIF2α and CRT proteins were detected using Western blotting. (**C**) A549, LLC, A375 and B16F10 cells were treated with 10 μM AsC or oxaliplatin for 24 h, the images of CRT were detected by the immunofluorescence. (**D**) Quantitative plot showing the secretory ATP content detected by ELISA. Data are shown as the mean ± SD, *n* = 3. * *p* < 0.05, ** *p* < 0.01, *** *p* < 0.001, **** *p* < 0.0001, “ns” indicates not significant.

Previous studies have shown that ER stress induced by ROS accumulation is the main cause of ICD, and is accompanied by the expression and secretion of numerous DAMPs, such as CRT and ATP [23,24]. To examine whether AsC can induce ER stress in lung cancer and melanoma cells and lead to ICD, this study examined the expression changes of ER stress-related proteins in lung cancer and melanoma cells after AsC treatment for different times or different concentrations. Western blotting analysis was performed to detect the levels of p-PERK, PERK, p-eIF2α, eIF2α and CRT in the lung cancer and melanoma cells. The results showed that with the extension of AsC treatment time or the increase in concentration, the expression of the majority ER stress-related proteins increased significantly. Interestingly, in LLC, we observed that as AsC treatment time increased, the expression of p-eIF2α tended to increase from 3 to 12 h, but then decreased. We also observed a similar phenomenon in B16F10 cells. The expression of p-eIF2α was significantly up-regulated at 3–24 h, and then returned to a lower expression state. Those phenomenon were not observed in A549 and A375 cells (Figure 5A). In contrast, the cellular heterogeneity was observed in the presence of oxaliplatin treatment. ER stress-related proteins were more significantly up-regulated in A549 and B16F10 cells under the treatment of oxaliplatin, while only some of molecules were up-regulated in LLC and A375 cells (Figure 5B). Furthermore, we used immunofluorescence experiments to detect the ectopic expression changes of CRT protein in the cells under the treatment of AsC for 24 h. The results showed that AsC increased the expression of CRT protein on the cell membrane (Figure 5C). In addition, using ELISA experiments, we found that the amount of ATP released in the cell supernatant increased significantly under the treatment of AsC for 24 h (Figure 5D). The above results indicate that AsC could induce ER stress in tumor cells, and the secretion of DAMPs leads to immunogenic death of lung cancer and melanoma cells.

**Figure 6:**
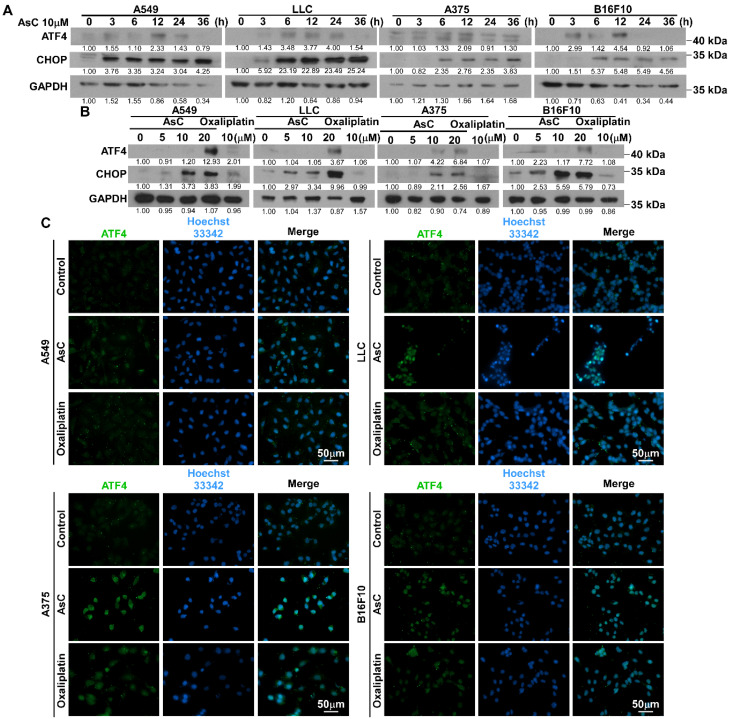
AsC activates the ATF/CHOP signaling pathway. (**A**) A549, LLC, A375 and B16F10 cells were treated by AsC at indicated concentrations for different times (0, 3, 6, 12, 24, 36 h). Levels of ATF4 and CHOP proteins were detected using Western blotting. (**B**) A549, LLC, A375 and B16F10 cells were treated with 10 μM AsC or oxaliplatin for 24 h. Levels of ATF4 and CHOP proteins were detected using Western blotting. (**C**) A549, LLC, A375 and B16F10 cells were treated with 10 μM AsC or oxaliplatin for 24 h, the images of ATF4 were detected by the immunofluorescence.

As a transcription factor that activates genes, ATF4 plays a key role in important biological processes such as autophagy, protein folding, amino acid metabolism, redox balance and apoptosis [25,26,27,28]. Previous studies have shown that ER stress-induced eIF2α phosphorylation activates the transcription and translation levels of ATF4, subsequently increasing glutathione biosynthesis and protecting cells from oxidative stress [29]. Interestingly, ER stress can also induce cell death through the ATF4/CHOP pathway [30]. In this study, our experiments observed that after treated with AsC for different times or different concentrations, the levels of CHOP was increased in both time- and concentration-dependent manner. However, the changes in ATF4 protein levels were not significant compared to those of p-eIF2α (Figure 6A,B). Furthermore, when cells were treated with 10 μM AsC for 24 h, ATF4 protein was more localized in the nucleus (Figure 6C). These results indicated that AsC mainly worked by promoting the entry of ATF4 into the nucleus, and the entry of ATF4 into the nucleus was an important sign of its activation. The above experimental results showed that AsC could induce ER stress, activate the PERK/eIF2α/ATF4/CHOP signaling pathway and cause ICD in lung cancer and melanoma cells.

### 2.6. AsC Promotes DC Maturation by Upregulating ROS Production

**Figure 7:**
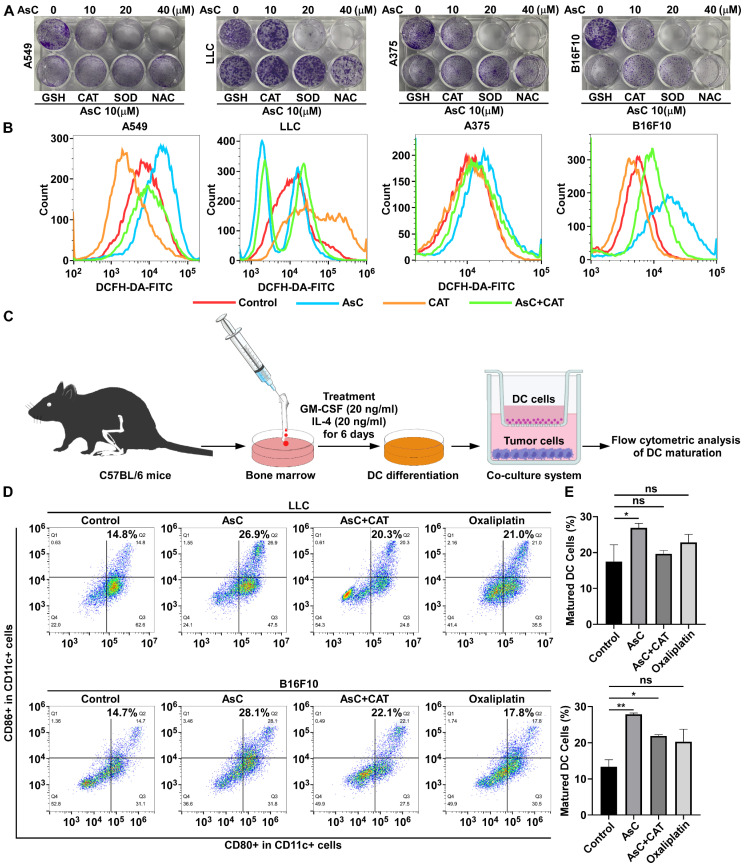
AsC promotes dendritic cell (DC) maturation by upregulating ROS production. (**A**) Clone formation experiments were performed to detect the effect of AsC on cell growth when A549, LLC, A375 and B16F10 cells were treated with or without the ROS scavenger, 2.5 mM Glutathione (GSH), 100 μ/mL Catalase (CAT), 100 μ/mL Superoxide dismutase (SOD) and 2.5 mM N-acetyl cysteine (NAC) for 10 days. (**B**) A549, LLC, A375 and B16F10 cells were treated with AsC (10 μM) with or without CAT (100 μ/mL) for 24 h. ROS were detected by flow cytometry. (**C**) The scheme of murine s DCs isolation, differentiation, and maturation analysis. (**D**) After the AsC treated with or without CAT LLC or B16F10 cells for 6 h, then co-cultured with immature DCs for another 24 h. The CD11c, CD80 and CD86 positive cells were detected by the flow cytometry. (**E**) Quantification of mature DCs following the CD11c, CD80 and CD86 staining. *n* = 3, * *p* < 0.05, ** *p* < 0.01, “ns” indicates not significant.

To further clarify the relationship between the antitumor activity of AsC and the production of ROS, we used four different ROS scavengers (Glutathione, GSH; Catalase, CAT; Superoxide dismutase, SOD and N-acetyl cysteine, NAC) to observe their effects on AsC’s inhibition of cell growth. The results showed that CAT and SOD had obvious effects and could weaken the inhibitory action of AsC on cell growth (Figure 7A). Additionally, AsC-stimulated ROS generation could be markedly reversed in the cells co-treated with CAT in most cases detected by flow cytometry (Figure 7B).

Calreticulin exposure is an important marker of ICD of tumor cells, providing immune cells with an “eat me” immunogenic signal [31,32]. In addition, the release of ATP from dead cells is a major feature of ICD. After binding to the purinergic receptor P2Y2, it acts as a “find me” signal for DC precursors, thereby promoting the recruitment of myeloid cells to active ICD sites [33,34]. ATP can function as a danger signal, promoting the maturation and differentiation of DCs. This results in increased expression of CD80 and CD86 in DCs, thereby strengthening T cell activation signals [35].

In order to further study the regulatory effect of AsC on DC maturation, based on a previous study [20], we isolated bone marrow from the femurs of C57BL/6 mice, used cytokines granulocyte-macrophage colony-stimulating factor (GM-CSF) and interleukin 4 (IL4) to induce their differentiation into DCs for 6 days, and then co-cultured them with tumor cells from different treatment groups for 24 h (Figure 7C). Flow cytometry was used to detect the proportion of mature DCs (CD11c^+^, CD80^+^, CD86^+^). As shown in Figure 7D,E, in LLC cells, the proportion of mature DCs increased from 14.8% in the control group to 26.9% in the AsC-treated group, and CAT partially reversed the induction of AsC on DCs. A similar phenomenon was observed in B16F10 cells (Figure 7D,E). These data indicated that AsC promoted DC maturation in a ROS-dependent manner.

## 3. Discussion

Natural products and their derivatives are important sources of antitumor drugs and can provide more new inhibitors or new framework active compounds. According to statistics, 62 of the 185 antitumor small molecule drugs approved from 1981 to 2019 were derived from natural products, accounting for 33.5% of the total [13]. The natural product is a compound of a twelve-membered or thirteen-membered macrocyclic ether and a tetracyclic (6/5/6/5) structure, which is a macrocyclic alkaloid. This type of compound was first discovered in 2001 [36]. Subsequent research results found that this type of compound exhibits significant antitumor activity. For example, GKK1032B induces apoptosis in human osteosarcoma MG63 cells by activating the Caspase signaling pathway [37]. Pyrrocidines A and B compounds are effective in human early childhood tumors. Myeloid leukemia HL-60 cells are cytotoxic [38]. Pyrrocidines A activates the Caspase-3/PARP signaling pathway in a ROS-independent manner to induce apoptosis of HL-60 cells [39]. Embellicines A and B have killing effects on leukemia cells K562 [40]. However, there are few reports of these compounds inducing ICD of tumor cells. In this study, we report the novel structure marine compound, AsC, inhibits lung cancer and melanoma growth in vivo and in vitro. Mechanistically, we first found that AsC could induce ER stress and increase ROS generation, which in turn upregulated DAMPs molecular expression and caused ICD. Furthermore, AsC promoted the maturation of DCs in vitro and improved the infiltration CD8^+^ T cells in tumors in vivo, which altered the tumor microenvironment eventually.

Advantages of marine natural products in drug development, marine microbial secondary metabolites have three important advantages compared with traditional animal and plant products. First, the use of large-scale fermentation projects can quickly and massively obtain the required compounds and solve the problem of drug sources; secondly, without the need for large-scale mining to avoid damage to the ecological environment; finally, by studying compound synthesis routes, adjusting fermentation conditions and the activity of key metabolic enzymes, it is possible to obtain more structurally novel compounds, which will be more conducive to improving the utilization of marine resources [41]. The compound AsC in this article was isolated from the secondary metabolites of marine endophytic fungi. There were four other structurally similar compounds discovered at the same time as AsC. Our previous studies compared the antitumor activities of these five compounds in various cancer models, including lung cancer, colon cancer, glioma, prostate cancer, melanoma and breast cancer. It was found that AsA and AsC showed more prominent activity in these six cancer models and had a higher acquisition rate [14]. Moreover, our previous research found that AsA blocks the cell cycle through the ROS-dependent Akt/Cyclin D1/Rb signaling pathway [42]. In this article, we also reveal the antitumor activity and biological effects of AsC, which makes AsC an attractive potential antitumor candidate compound.

The relationship between ROS, tumors and tumor immunity is close and complex [43]. ROS exerts both antitumor [44,45,46,47] and potential tumor-promoting effects [48,49,50,51], and the same is true for tumor immunity. This may depend on the concentration, location, type and microenvironment of ROS. Due to the abnormal metabolism of tumor cells and continued activation of important signaling pathways, cancer cells have higher ROS levels than normal cells. However, excessive ROS accumulation is harmful to cells, such as destroying cell membranes, proteins, lipids and nucleic acids, and even kills cells. Therefore, tumor cells are more dependent on antioxidant mechanisms than normal cells [52]. This makes the development of ROS inducers potentially an effective strategy against tumors, such as β-lapachone [53]. The transcription factor NF-E2 p45-related factor 2 (NRF2; encoded by NFE2L2) and its principal negative regulator, the E3 ligase adaptor Kelch-like ECH-associated protein 1 (KEAP1) plays a key role in maintaining redox [54]. Our results show that AsC seems to have good inhibitory activity against Keap1-mutated cells, including PC9, H460 and A549, with IC_50_ 4.98, 8.93 and 10.25 μM, respectively. The IC_50_ of Keap1 wild-type cells H1703, H1975 and H1299 are 7.29, 10.50 and 15.05 μM, respectively. Although this difference is weak, it also indicates to a certain extent that AsC has a strong inhibitory effect on NRF2/Keap1 mutant cells. This type of cell relies on its own powerful antioxidant system to maintain its growth, which in turn shows that these cells are vulnerable to ROS. This reminds us that if AsC is combined with an NRF2 inhibitor, it is possible to obtain better therapeutic effects.

The Unfolded Protein Response (UPR) is a cellular stress response specifically related to the endoplasmic reticulum (ER) stress. It serves three primary physiological functions: firstly, it halts protein translation to restore normal cellular functions; secondly, it reduces the accumulation of misfolded proteins; and thirdly, it activates specific signaling pathways to enhance the production of molecular chaperones that aid in protein folding. The UPR’s role is to mend cellular damage and maintain the survival of organisms [55,56]. However, prolonged UPR can lead to cell death via the PERK/eIF2α/ATF4/CHOP signaling pathway [10,57]. Studies indicate that cancer cells may evade drug-induced death by upregulating protective proteins like the binding-immunoglobulin protein (Bip), making UPR inducers in combination with protein synthesis inhibitors a novel anticancer therapy [58]. Additionally, this study observed that AsC treatment inhibited the expression of p-eIF2α and ATF4 in lung cancer and melanoma cells after 36 h. However, the mechanism needs further investigation, which might pave the way for new anticancer therapies.

With the discovery of immune checkpoints, immunotherapy has emerged as a critical modality for cancer treatment alongside surgery, chemotherapy and targeted therapy. However, only a limited subset of patients benefits from immune checkpoint therapy. A primary reason is the “immune desert” state of most patients, characterized by minimal immune cell infiltration within the tumor microenvironment. Thus, the development of drugs that boost immune cell infiltration represents a promising avenue. Research indicates that inducing ICD in tumor cells is a key strategy to enhance tumor immunity. Numerous drugs and natural substances have been identified to trigger ICD in tumor cells [2,59]. For instance, doxorubicin from Streptomyces can initiate the exposure of CRT, heat shock proteins 70 and 90 (HSP70/HSP90) and the release of IFN-γ, fostering DC maturation [60,61]. In addition, capsaicin extracted from peppers can also induce ICD, promoting ROS production, leading to ER stress, CRT exposure and release of HMGB1 and ATP [62,63,64]. Oxaliplatin, a prevalent chemotherapy drug, has been found to elevate ICD biomarkers in liver cancer cells and, in vivo, to increase mature DCs and CD8^+^ T cells while decreasing regulatory T (Treg) cells. Moreover, combining oxaliplatin with immune checkpoint inhibitors has demonstrated a synergistic effect in cancer treatment [4]. In this study, we found that AsC can successfully induce ICD in lung cancer and melanoma cells, thereby promoting the maturation of DCs and increasing the infiltration of CD8^+^ T cells into tumors.

In the realm of clinical tumor immunotherapy, the targeting of the Programmed death 1 (PD-1)/PD-L1 pathway has emerged as one of the most triumphant and prevalent approach [65,66]. While antibody drugs remain the mainstream, the universal application of drugs has been hindered to some extent due to their high price and special conditions of use. Consequently, there is a growing shift towards developing small molecule drugs for antitumor immunotherapy [67]. Research has revealed that various small molecule drugs can modulate PD-L1 expression, including gefitinib and doxorubicin, or can disrupt the PD-1/PD-L1 pathway signaling by targeting their binding site, with compounds like CA-170 and BMS-202 [68]. Notably, natural products, such as AsC, with their larger molecular weights and complex structures, are hypothesized to have superior potential in blocking protein–protein interactions, making them promising candidates for small molecule immune checkpoint inhibitors.

In summary, we discovered the antitumor activity of the 13-membered macrocyclic alkaloid structure compound AsC in in vivo and in vitro. At the same time, this study also revealed the antitumor mechanisms of AsC. AsC could upregulate cellular ROS levels, induce ER stress in tumor cells, activate the PERK/eIF2α/ATF4/CHOP signaling pathway, cause tumor ICD and change tumor immunity microenvironment eventually. This study not only expands our understanding of the antitumor effects of 13-membered macrocyclic alkaloid compounds, but also takes an important step towards promoting AsC as an antitumor lead compound.

## 4. Materials and Methods

### 4.1. Preparation of AsC

AsC was prepared and purified from mangrove endophytic fungus Ascomycota sp. CYSK-4 as previously reported, and its structure was identified by interpretation of spectral data (MS, 1H NMR, 13C NMR, 2D NMR) and X-ray single crystal diffractive technique) [14]. The compound was dissolved in 99.9% dimethyl sulfoxide (DMSO) at a concentration of 10 mM as stock solution and diluted according to experimental requirements when used.

### 4.2. Cell Culture

Human lung cancer cell lines A549, H1975, H460, H1299, H1703, PC9 and human melanoma cell lines A375 were obtained from the cell bank of the Shanghai Institutes of Biological Sciences (Shanghai, China) or from Fu Erbo Biotechnology Co., Ltd. (Guangzhou, China). Mouse cancer cell lines LLC, Hepa1-6, MC38, B16F10, CT26 and 4T1 were purchased from ATCC. Cells were cultured in Dulbecco’s modified Eagle’s medium (DMEM) (Invitrogen, Carlsbad, CA, USA) supplemented with 5% fetal bovine serum (Hyclone, Logan, UT, USA), 2 mM L-glutamine, 100 mg·mL^−1^ streptomycin and 100 units·mL^−1^ penicillin (Invitrogen, Carlsbad, CA, USA). The cultures were maintained at 37 °C in a humidified atmosphere of 5% CO_2_.

### 4.3. Cell Viability Assay

Cell viability was determined by 3-(4,5-dimethylthiazol-2-yl)-2,5-diphenyl tetrazolium bromide MTT) reagent (Genview, Houston, TX, USA) assay as previously described [42].

### 4.4. Clonogenic Assay

Tumor cells were plated in 24-well plates, A549 and B16F10 cells at a density of 1000 cells/well, A375 and LLC cells at a density of 2000 cells/well. After cells were attached for 3 days, AsC or oxaliplatin at indicated concentrations was added to plates. DMSO served as the control vehicle. The cells were then fixed with methanol and stained with 0.1% crystal violet when AsC or oxaliplatin treatment for 7 days. Colonies were counted by Image J 1.50i software NIH, Bethesda, MD, USA).

### 4.5. Cell Apoptosis Analysis by Flow Cytometry

Apoptotic cell death was determined by flow cytometry analysis using Annexin V-APC apoptosis detection kit (Sungene Biotech, Tianjin, China). After 24 h individual AsC or oxaliplatin treatment, cells were collected, washed with cold PBS, suspended in 500 μL of binding buffer and stained with 5 μL of Annexin V-APC and propidium iodide respectively. The cells were mixed gently, incubated in the dark for 30 min. The samples were analyzed with a FACS (Beckman Coulter, CA, USA).

### 4.6. Western Blotting Analysis

The cells were seeded in 60 mm diameter plates at 1 × 10^6^ cells per well. After incubation for 24 h, the cells were treated with 10 μM AsC for specified time or treated with AsC or oxaliplatin at the indicated concentrations for 24 h. The cells were harvested and lysed after treatment then lysates were probed with primary antibodies and horseradish peroxidase (HRP) (1:2000; Bio-Rad Laboratories, Inc., Hercules, CA, USA) conjugated secondary antibodies. Primary antibodies including p-PERK (1:500, RRID: AB_2935416), PERK (1:500, RRID: AB_2879521), eIF2α (1:500, RRID: AB_2096489), CRT (1:500, RRID: AB_2880835), ATF4 (1:500, RRID: AB_2058600), CHOP (1:500, RRID: AB_2292610) and GAPDH (1:5000, RRID: AB_2107436) were purchased from Proteintech (Guangzhou, China). p-eIF2α (1:500, RRID: AB_3065164) was purchased from ImmunoWay Biotechnology (Jiangsu, China).

### 4.7. Chemicals and Fluorescent Probes for Studying ROS Generation

A549, LLC, A375 and B16F10 cells were plated on 24 mm dishes at 2 × 10^4^ cells per well and cultured for 24 h, and then treated with AsC or oxaliplatin at the indicated concentrations for 24 h. Cells were incubated with 10 µM DCFH-DA (Biosharp Life Sciences, Hefei, China) without FBS at 37 °C with 5% CO_2_ for 30 min in the dark. Then, cells were centrifuged and resuspended in PBS before adding 10 µg/mL Hoechst33342 in DMEM without FBS and incubated again at 37 °C with 5% CO_2_ for 20 min in the dark. Cells were centrifuged and washed by PBS, and this process was repeated three times. Finally, DCF fluorescence (produced in the presence of ROS) was analyzed using an epifluorescence microscope equipped with a digital camera (Zeiss, Oberkochen, Germany). The wavelength Ex/Em = 355/465 nm (Hoechst 33342) and Ex/Em = 488/525 nm (FITC).

### 4.8. In Vivo Assay

All animal care and experimental procedures were approved by the Ethics Committee of the Institutional Animal Care and Use Committee (IACUC), Sun Yat-sen University (protocol code SYSU-IACUC-2021-B1229). C57BL/6J mice were inoculated subcutaneously (s.c.) with LLC or B16F10 (1 × 10^6^) cells in the right lower abdomen on day 0. Four randomized cohorts (LLC, *n* = 6; B16F10, *n* = 5) with a tumor size between 80 and 100 mm^3^ were administered the vehicle control (2%DMSO, 15%Tween 80, 83%physiological saline, i.p., TIW) or AsC (25 mg·kg^−1^, 50 mg·kg^−1^ i.p., TIW). The positive control group was given oxaliplatin (5 mg·kg^−1^ i.p., TIW). The tumor growth and regression were determined using volume as the readout. The volumes (V) were calculated using the following formula: V = π·6^−1^ × (length) × (width)^2^. Data were presented as the mean SD of mice in each group. Mice were humanely euthanized either at indicated times or when the tumor reached a maximum diameter of 15 mm, and the xenograft tumors were dissected and weighed. The tumor samples were cut for immunohistochemical (IHC) and flow cytometric analysis.

### 4.9. H&E Staining

The B16F10 tumor samples and mouse small intestine, renal and liver tissue were stained with Hematoxylin and Eosin (H&E) and observed using an inverted fluorescence microscope and output imaging system (Zeiss, Oberkochen, Germany).

### 4.10. Immunohistochemistry (IHC) Staining

The B16F10 tumor samples were used to analysis. Tumor samples were collected, fixed in 4% formalin for 24 h at 4 °C and anti-CD3, anti-PD-L1 were used for IHC. CD3 (1:200; RRID: AB_443425) and PD-L1 (1:200, RRID: AB_2687878) were purchased from Abcam (Cambridge, UK).

### 4.11. Tumor-Infiltrating Lymphocytes (TIL) Analysis by Flow Cytometric

The LLC and B16F10 tumor samples were collected and tissue was ground into individual cells then washed twice with pre-cooled PBS. Next 5 mL of red blood cell lysis solution (150 mM NH_4_CL, 10 nM KHCO_3_, 1 nM EDTA) was added for 5 min the washed once with pre-cooled PBS. Anti-CD45-FITC, anti-CD3-PE/Cyanine 7, anti-CD4-PE, anti-CD8-Brilliant Violet 421^TM^, anti-NK1.1-PE, anti-CD25-Brilliant Violet 421^TM^, anti-IL2-PE/Cyanine 7, anti-IFN-γ-PE and Ghost Dye™ Violet 510 were used for flow cytometry analysis. CD45 (RRID: AB_312972), CD3 (RRID: AB_1732068), CD4 (RRID: AB_312693), CD8a (RRID: AB_10897101), CD25 (RRID: AB_10895908), IL2 (RRID: AB_2561749) were purchased from BioLegend (San Diego, CA, USA). Ghost Dye™ Violet 510, NK1.1 (RRID: AB_2621804) and IFN-γ (RRID: AB_2621810) were purchased from Tonbo Biosciences (San Diego, CA, USA).

### 4.12. Immunofluorescence Staining

A549, LLC, A375 and B16F10 cells were plated on 24 mm dishes at 1 × 10^4^ cells per well for 24 h, and then treated with 10 µM AsC or oxaliplatin. After 24 h of AsC treatment, cells washed with 1 × PBS and fixed in 4% paraformaldehyde, followed by blockade with blocking buffer (10% BSA and 0.5% Triton X-100). The cells were incubated with the anti-CRT, anti-ATF4 primary antibody overnight at 4 °C. They were then washed three times with PBS, 10 min each time, incubated with secondary antibodies and shaken for 30–45 min at room temperature in the dark. Hoechest33342 was used for nuclear staining and images were captured using a Zeiss LSM 780 confocal microscope. CRT (1:200, RRID: AB_2880835) and ATF4 (1:200, RRID: AB_2058600) were purchased from Proteintech (Guangzhou, China).

### 4.13. JC-1 Mitochondrial Membrane Potential Assay

JC-1 mitochondrial membrane potential assay was performed as previously described [69]. Briefly, A549, LLC, A375 and B16F10 cells were plated on 24 mm dishes at 2 × 10^4^ cells per well and cultured for 24 h, and then treated with positive control (Carbonyl cyanide 3-chlorophenylhydrazone, CCCP), AsC or oxaliplatin at the indicated concentrations for 6 h. The mitochondrial membrane potential was assessed using JC-1 (Dojindo Molecular Technologies, Tokyo, Japan) staining according to the manufacturer’s protocols and previous reports. All images were obtained with a fluorescence microscope (Carl Zeiss, Oberkochen, Germany) with filter pairs of 561 nm/600 nm and 488 nm/561 nm. The fluorescence intensity was measured on Image J (Wayne Rasband, National Institutes of Health, Bethesda, MD, USA).

### 4.14. ATP Release Assay

ATP release assay according to the manufacturer’s instructions of the ATP assay kit (Beyotime, Shanghai, China). Briefly, A549, LLC, A375 and B16F10 cells were plated on 24 mm dishes at 2 × 10^4^ cells per well and cultured for 24 h, then treated with AsC or oxaliplatin at the indicated concentrations for 24 h. The supernatant was collected, and after centrifugation, the supernatant was added into the substrate solution. The luminescence was acquired using the SpectraMax L Microplate Reader (Molecular Devices, Silicon Valley, CA, USA).

### 4.15. In Vitro DC Maturation Assays

The DCs were isolated from the bone marrow of C57BL/6 mice following the previous study [20]. In brief, the mice were first euthanized and disinfected with 75% alcohol for 15 min. The tibia and femur were stripped, then the bone marrow was immediately flushed out using fresh 1640 culture medium. Thet were washed with PBS and incubated with red blood cell lysis solution for 5 min; the purified bone marrow cells were seeded in special 1640 medium containing GM-CSF (Novoprotein, Shanghai, China) (20 ng/mL) and IL-4 (20 ng/mL) in six-well culture plates. On day 3, half of the culture medium in plates was replaced with an equal volume of fresh culture medium. The immature DCs were obtained after 6 days of culture.

### 4.16. Detection of Intracellular ROS

The intracellular ROS levels were measured using a flow cytometer as previously described [42]. Following treatment, cells were harvested and washed with PBS and then suspended in serum-free DMEM containing 10 µM of 2′,7′-dichlorodihydrofluorescein diacetate (DCFH-DA) at 37 °C, 5% CO_2_ for 30 min. Cells were washed with PBS, and flow cytometry analysis was performed with an excitation wavelength of 488 nm and emission wavelength of 525 nm.

### 4.17. Statistical Analysis

All experiments were performed at least three times, and data were expressed as mean ± SD. Student’s *t*-test was used to compare the differences between two groups. We compared more than two groups with one-way ANOVA with Tukey’s post hoc test, the overall F test was significant (*p* < 0.05), and there was no significant variance in homogeneity. GraphPad Prism version 5.0 (GraphPad Software, San Diego, CA, USA) was employed to conduct the statistical analysis. All statistical tests were two-sided, and *p* < 0.05 was considered statistically significant. The data and statistical analyses comply with the recommendations on experimental design and analysis in pharmacology.

### 4.18. Materials

Hoechest33342 (#HY-15559, CAS Number: 23491-52-3) and oxaliplatin (#HY-17371, CAS Number: 61825-94-3) were purchased from MedChemExpress (Shanghai, China), N-Acetyl-L-cysteine (NAC; #A7250, CAS Number: 616-91-1), 2′,7′-dichlorodihydrofluorescein diacetate (DCFH-DA; #287810, CAS Number: 4091-99-0) and Dimethyl sulfoxide (DMSO; #D2650, CAS Number: 67-68-5) were purchased from Sigma-Aldrich (St. Louis, MO, USA). Carbonyl cyanide 3-chlorophenylhydrazone (CCCP, #C6700, CAS Number: 555-60-2) and Superoxide Dismutase (SOD; # S8410, CAS Number: 9054-89-1) were purchased from Beijing Solarbio Science and Technology Co. (Beijing, China). Catalase (CRT, #MB3116, CAS Number: 9001-05-2) and Glutathione (GSH; #571190-30-2, CAS Number: 70-18-8) were purchased from Dalian Melun Biotechnology Co. (Dalian, China). Annexin V-APC apoptosis analysis kit (#AO2001-11P-H) was purchased from Sungene Biotech (Tianjin, China). ATP assay kit (#S0026) was purchased from Beyotime (Shanghai, China).

## Data Availability

The data presented in this study are available on request from the corresponding author.

## Supplementary Materials

The following supporting information can be downloaded at: https://www.mdpi.com/article/10.3390/md21120600/s1, Figure S1: MTT assay to detect the cell viability of oxaliplatin in human cancer cells (A549, A375) (A) and mouse cancer cells (LLC, B16F10) (B); Figure S2: H&E staining to observe the damage to mouse small intestine, renal and liver tissue treated with AsC and oxaliplatin; Figure S3: Flow cytometry analysis of the effects of AsC and oxaliplatin treatment on the proportion of (A) CD4^+^ T and (B) CD8^+^ T cells, GzmB^+^ cells and IL2^+^ T cells in LLC; Figure S4: Flow cytometry analysis of the effects of AsC and oxaliplatin treatment on the proportion of (A) CD4^+^ T and (B) CD8^+^ T cells, IFN-γ^+^ T cells and IL2^+^ T cells in B16F10; Figure S5: Flow cytometry analysis of the effects of AsC and oxaliplatin treatment on the proportion of NK cells in LLC.
