# Supplementary material for: Ascomylactam C Induces an Immunogenic Cell Death Signature via Mitochondria-Associated ER Stress in Lung Cancer and Melanoma
Source: Mar Drugs. 2023 Nov 21;21(12):600. doi: 10.3390/md21120600 (PMC10744434; doi:10.3390/md21120600)
Supplement: Supplementary file 1 [file marinedrugs-21-00600-s001.zip › marinedrugs-2699911-supplementary.pdf]

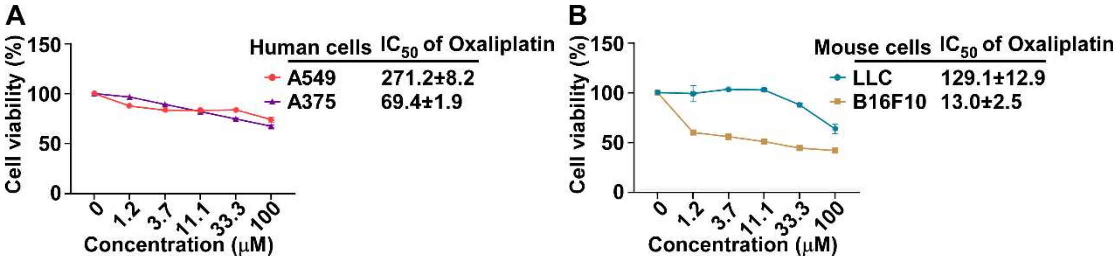

Figure S1. MTT assay to detect the cell viability of oxaliplatin in human cancer cells (A549, A375) **(A)** and mouse cancer cells (LLC, B16F10) **(B)**.

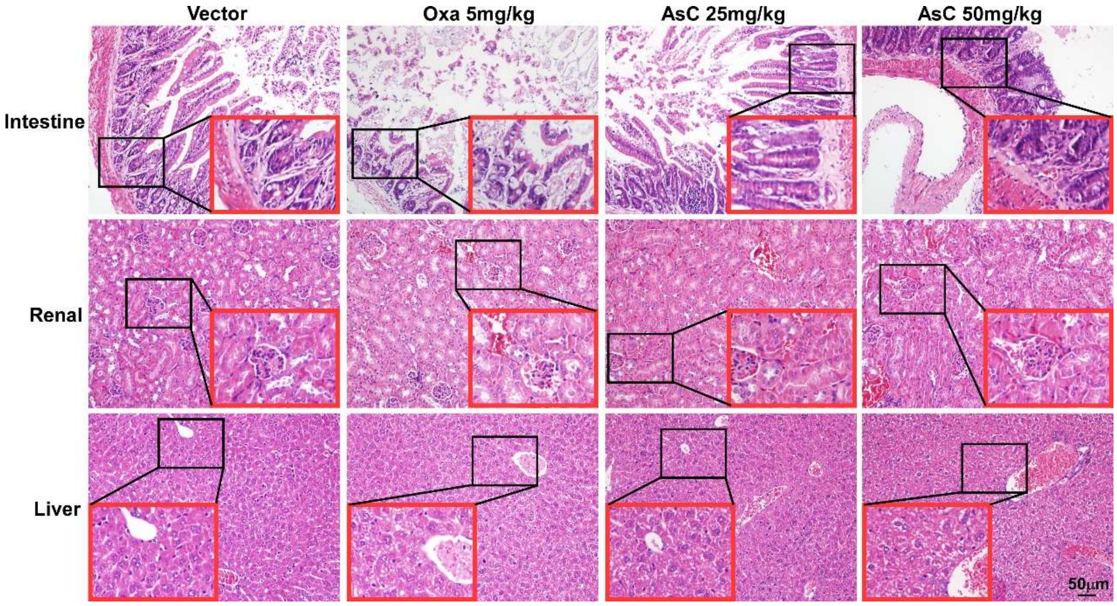

Figure S2. H&E staining to observe the damage to mouse small intestine, renal and liver tissue treated with AsC and Oxaliplatin.

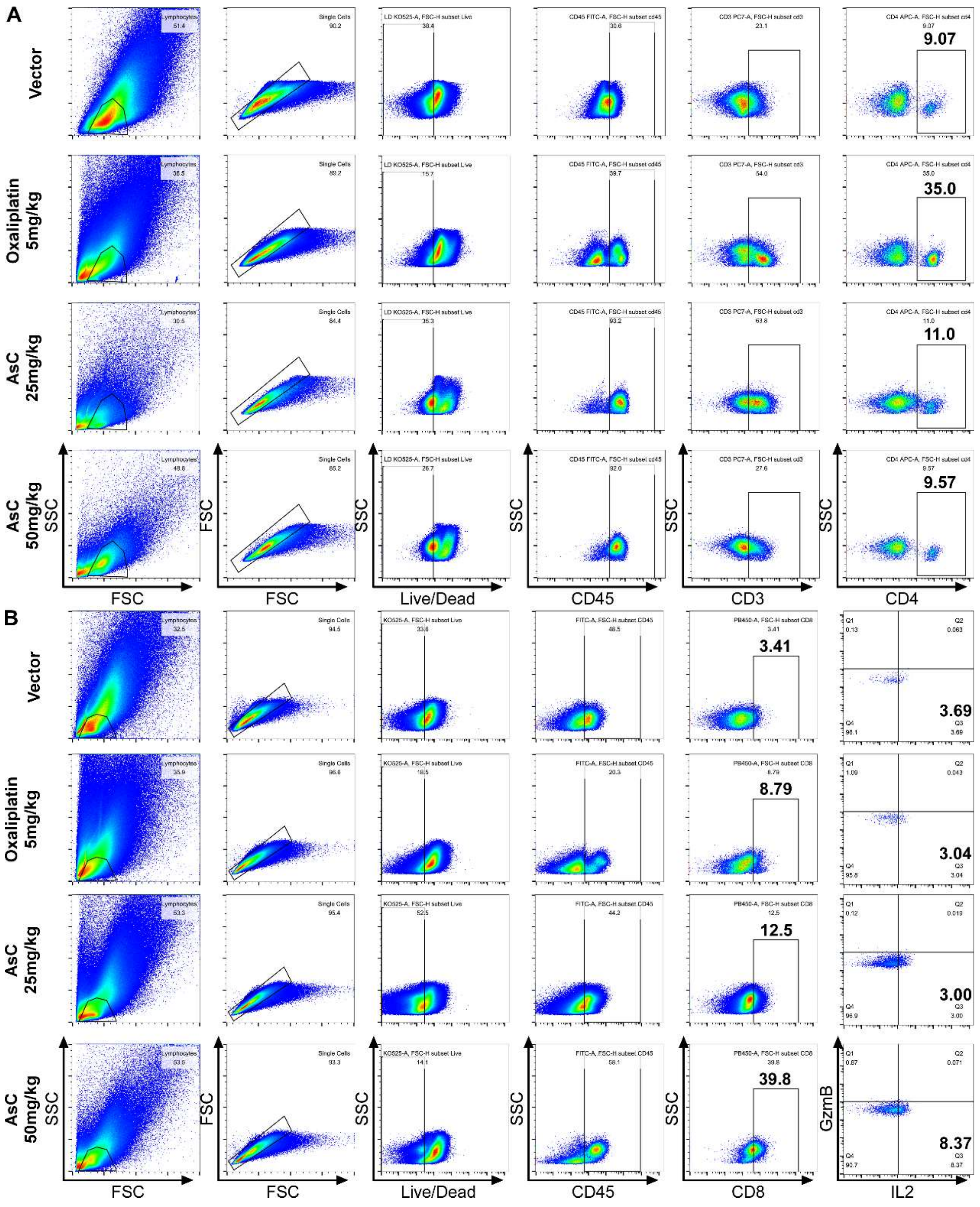

Figure S3. Flow cytometry analysis of the effects of AsC and Oxaliplatin treatment on the proportion of (A) $CD4^+$ T and (B) $CD8^+$ T cells, $GzmB^+$ T cells and $IL2^+$ T cells in LLC.

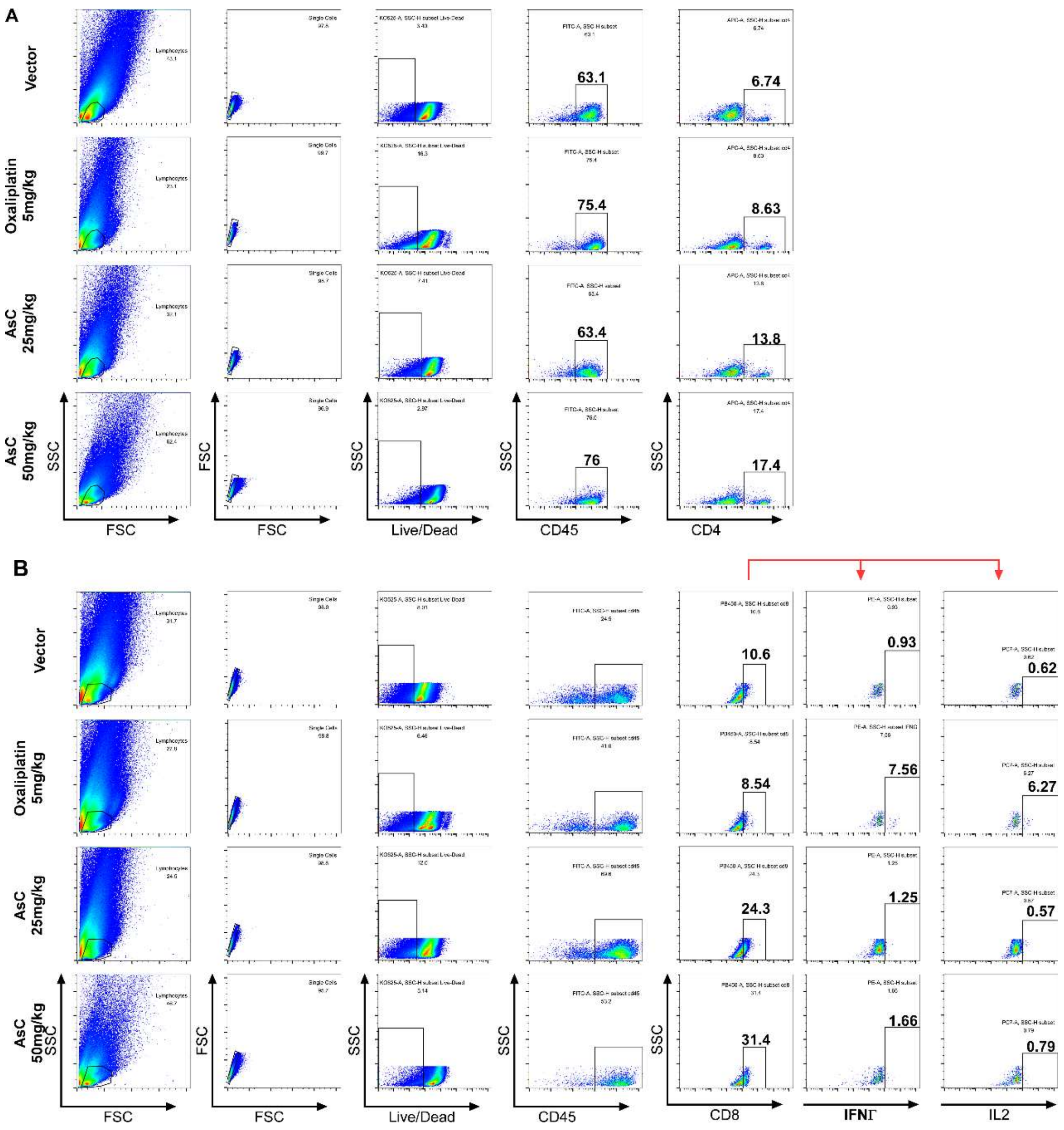

Figure S4. Flow cytometry analysis of the effects of AsC and Oxaliplatin treatment on the proportion of (A) $CD4^+$ T and (B) $CD8^+$ T cells, IFN-γ^+ T cells and $IL2^+$ T cells in B16F10.

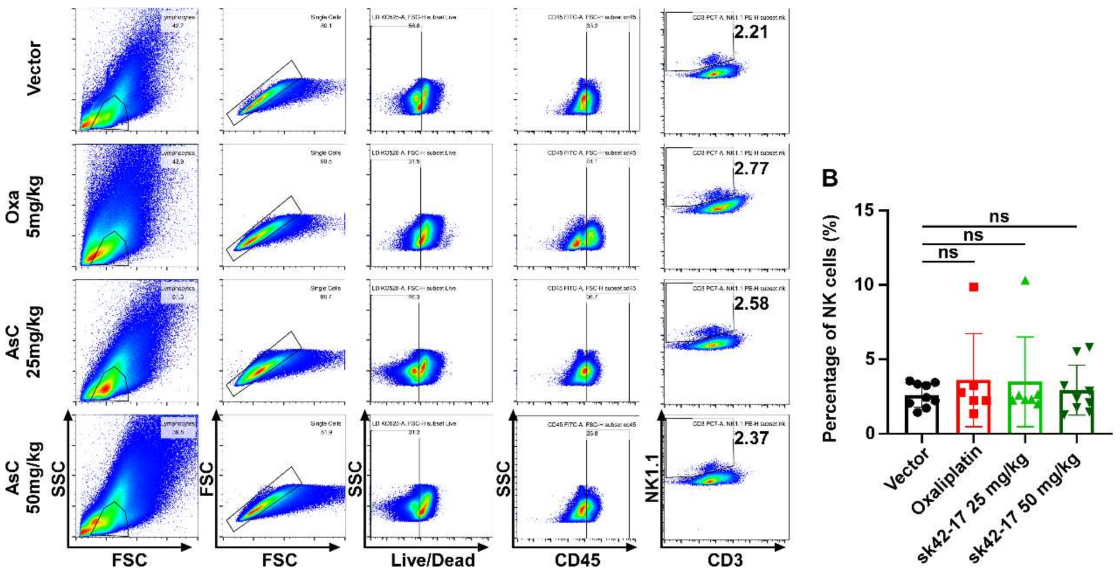

Figure S5. Flow cytometry analysis of the effects of AsC and Oxaliplatin treatment on the proportion of NK cells in LLC.
